# Pain medication misuse in the South African spinal cord injury context

**DOI:** 10.4102/hsag.v29i0.2377

**Published:** 2024-01-31

**Authors:** Mokgadi K. Mashola, Elzette Korkie, Diphale J. Mothabeng

**Affiliations:** 1 Department of Physiotherapy, Faculty of Health Sciences, University of the Witwatersrand, Johannesburg, South Africa; 2 Department of Physiotherapy, Faculty of Health Sciences, University of Pretoria, Pretoria, South Africa

**Keywords:** analgesics, opioid, pain, pain medication misuse, spinal cord injury

## Abstract

**Background:**

Pain after spinal cord injury (SCI) is debilitating and has been reported to be difficult to treat, despite pharmacological interventions. Pain medication misuse (PMM) and associated individual factors among people with spinal cord injury (PWSCI) are scarce.

**Aim:**

To determine PMM and the associated factors in PWSCI.

**Setting:**

Homes of community-dwelling manual wheelchair users with SCI in South Africa.

**Methods:**

Community-dwelling PWSCI (*n* = 122) were consecutively sampled and the Pain Medication Questionnaire (PMQ) was used to determine PMM. Descriptive statistics, Fisher’s exact test, independent t-tests, and simple linear regression tests were performed using SPSS v27. Testing was conducted at the 0.05 level of significance.

**Results:**

Eighty-five per cent of the participants reported the presence of pain and 48.1% of them used pain medication. Forty-four percent of people who used pain medication scored ≥ 30, indicative of serious aberrant drug-taking behaviours. Opioids were mainly used for neuropathic pain and in combination with other types of medications such as anticonvulsants and non-steroidal anti-inflammatories (44.0%). Pain severity and the type of pain medication were found to be predictors of PMM (*p* < 0.01 respectively).

**Conclusion:**

Pain relief after SCI remains difficult to achieve, with an evident high risk of PMM, which may lead to long-lasting side effects, dependency, or overdose.

**Contribution:**

This study has shown the need for the assessment of the potential risk of dependency before prescribing pain medication, particularly opioids to PWSCI.

## Introduction

Spinal cord injury (SCI) gives rise to various secondary health conditions such as pain and pressure ulcers, among others (Krause, Clark & Saunders 2015). Pain estimates in people with SCI (PWSCI) are as high as 94% and tend to be more severe than in the able-bodied population (Mashola & Mothabeng 2019; Pilusa, Myezwa & Potterton 2021). Increased pain severity is linked to greater pain medication dependency and poor quality of life (Krause et al. 2015). Various factors such as age, gender, mental health, socio-economic status, and sensitivity to drug effects may contribute to pain medication misuse (PMM) (Sehgal, Manchikanti & Smith 2012; St Marie 2019). Medication misuse is defined as the ‘use of a medication (for a medical purpose) other than as directed or as indicated, whether wilful or unintentional and whether harm results or not’ (Katz et al. 2007:650).

Pain medication misuse is an important concept to be mindful of as pharmacological interventions are often prescribed in the SCI population (Krause et al. 2015; Sehgal et al. 2012). People with SCI have mainly been prescribed paracetamol, non-steroidal anti-inflammatories (NSAIDs), acetaminophens, and opioids for nociceptive pain (Mahnig et al. 2016; Tsai et al. 2021), as well as anticonvulsants and antidepressants for neuropathic pain (Davari et al. 2020). An estimated 37% of people without SCI and living with chronic pain misuse pain medication, and PWSCI have 7.97 greater odds of developing opioid misuse than their able-bodied counterparts (Clark, Cao & Krause 2017; Graupensperger et al. 2019). People with SCI are a vulnerable group that has demonstrated aberrant drug-use behaviour. Aberrant drug-use behaviour is considered the type of behaviour that falls outside the boundaries of the treatment agreed upon with prescribers (Clark et al. 2017). People with chronic pain who receive higher doses of pain medication are at risk of overdose (Sehgal et al. 2012).

Spinal cord injury is a long-lasting condition, with the potential for pain to be present throughout the individual’s life. People with SCI therefore have increased chances of using pain medication for a prolonged time and thus have a greater risk of PMM, especially if they have low levels of education, increased pain severity, and greater limitations in activities of daily living (Krause et al. 2015). There is a need for non-pharmacological interventions to be considered to prevent dependency on pain medication. Therapeutic interventions such as physiotherapy are recommended as the first-line treatment of pain (Van Straaten et al. 2017), yet pharmacological interventions remain the mainstream choice of pain management in the SCI population, despite PWSCI reporting that pharmacological treatments provide very minimal pain relief (Celik et al. 2013; Guy et al. 2014). The substance abuse and mental health services administration (SAMHSA) recommends that non-pharmacological therapy be preferred for chronic pain, and healthcare providers (HCPs) should consider opioid therapy only if the expected benefits for both pain and function are anticipated to outweigh the risks to the patient using the opioids (SAMHSA 2016). This study aimed to determine PMM and its associated factors in PWSCI in South Africa. The specific objectives were to determine the pain characteristics and type, describe the type of medication prescribed to manage pain as well as the prevalence of PMM in this population.

## Research methods and design

### Study design and participants

This study used a quantitative correlational study design and is part of an umbrella study by the authors (Mashola et al. 2021) which aimed to determine the presence of pain in PWSCI and develop a guided pain self-management intervention framework. The study setting was the participants’ homes or private offices that were within a driving range of a 500 km radius from their discharging hospital situated in Gauteng, South Africa.

### Population, sample, and sampling

The authors used a consecutive sampling method where all adult PWSCI were enrolled as they consented to participate in the study. Consenting participants were excluded if they were readmitted to the hospital, moved residences beyond the specified driving range at the time of data collection, or did not honour their appointments.

The events per variable (EPV) approach was used to derive the sample size in the main study (Peduzzi et al. 1996; Vittinghoff & McCulloch 2007). A minimum of 35% of manual wheelchair users were hypothesised to experience pain after SCI (Dijkers, Bryce & Zance 2009), with eight variables hypothesised to be associated, namely age, gender, type of occupation, years living with SCI, neurological level of injury (NLI), completeness of injury, pectoralis minor muscle length, and the presence of scapular dyskinesis. The EPV > 5 × 8 = 40, and 40/0.35, result in a sample size of 115.

### Data-collection tools

The authors used a sociodemographic and injury profile sheet to document the participants’ demographic information such as age and gender, SCI profile such as type of injury, and pain characteristics. Pain characteristics included the number of painful areas, the location of the pain, as well as type and nature of the pain. The authors coded the painful areas from the most painful area (P1) to the least painful area (P5). The severity of pain was determined using the Numeric Rating Scale (NRS), an 11-point Likert scale where ‘0’ = no pain and ‘10’ = the most intense pain that was experienced in the last 24 h. The NRS has adequate construct and content validity in the SCI population (*r* = 0.38) (Bryce et al. 2007; Dijkers 2010). The 10-item Douleur Neuropathique 4 Questions (DN4) questionnaire was used to classify the type of pain as neuropathic pain or not and has shown high inter-rater reliability values of between 0.70 and 0.96 (Bouhassira et al. 2005). The 26-item Pain Medication Questionnaire (PMQ) was used to assess the risk for PMM in participants who reported pain (Clark et al. 2017). Questions include asking PWSCI if they believe they would feel better with a higher dosage of the pain medication and family and friend’s involvement such as borrowing their pain medication. Scores range from 0 to 104, with scores between 25 and 29 suggesting a risk of future problematic use. Scores ≥ 30 suggest a more serious aberrant drug-taking behaviour and this cut-off was also used to define the risk of PMM in this study. The PMQ has adequate construct and content validity, with a Cronbach’s alpha of 0.73 and test–retest reliability (*r* = 0.84) (Clark et al. 2017). Although validated in the SCI population, the above-mentioned questionnaires are yet to be validated in the South African context.

### Data-collection procedure

The first author perused the databases of the five consenting rehabilitation institutions from May 2018 to December 2018 to identify potential participants. Each potential participant was then contacted via telephone by the first author to invite them to participate in the study in January 2019. Once verbal consent was obtained telephonically, the first author travelled to the homes of all the potential participants from February 2019 to March 2020, and an informed consent form was signed on the day of the visit. Although all questionnaires used in this study were self-reported, they were researcher-administered to ensure consistency and make sure that the participants understood the questions. There were no language challenges encountered during the administration of the questionnaires (Mashola, Korkie & Mothabeng 2022).

### Data analysis

The first author captured the raw data on hard-copy capture sheets and transferred it to Microsoft Excel (version 2016) during data management. The SPSS v27 was used to analyse the data. Descriptive statistics including the mean, standard deviation, data frequencies, and percentages were used to report the descriptive statistics. Fisher exact tests were used to determine associations between PMM and demographic details while simple linear regression tests were conducted to determine the predictors of PMM. Testing was conducted at the 0.05 level of significance and 95% confidence intervals. Bootstrapping was performed during analysis to correct for optimism as recommended by Vittinghoff and McCollough (2007), and the bootstrap results are based on 1000 bootstrap samples.

### Ethical considerations

This study is registered with the South African National Health Research Database (reference GP201806005) and received ethical approval from the Faculty of Health Sciences Research Ethics Committee of the University of Pretoria, South Africa (approval number 125/2018). The participating hospitals granted permission to peruse their databases and written informed consent was obtained from all the participants in this study. Only the lead author was privy to the participants’ real names, which are not used in this paper to ensure confidentiality. Furthermore, the participants had the autonomy to discontinue at any point of the data-collection duration without any repercussions. All data from this study will remain stored for no less than 15 years as per the data safekeeping requirements of the research ethics committee.

## Results

### Socio-demographic information

A total of 122 PWSCI consented and were included in the study. Most of the participants were male (68%), between the ages of 31 years and 45 years (53.1%), black (85.2%), and were living with SCI for between 1 year and 5 years (50.8%). The mean standard deviation (SD) age of participants was 39.7 (11.1) years and the mean age (SD) when injured was 32.6 (10.7) years, while the mean years living with SCI was 7.1 years (SD 7.1). Most of the participants resided with their families (50.8%), in the township area (45.9%), were unemployed (59.0%), and relied on the government disability grant (47.5%). The common level of SCI was between T6 and T12 (73.8%) and the injuries were mostly complete (76.2%). Motor vehicle accidents were the most common cause of SCI (41.0%).

### Pain characteristics

Eighty-five per cent of participants reported pain and the type of pain experienced was mainly neuropathic pain (62.5%). The pain was mostly in one area (48.4%) and could be spread for up to five painful areas. The two most painful areas (P1 and P2 respectively) were neuropathic pain (73.1% and 23.1% respectively), while the third and fourth painful areas (P3 and P4) were more nociceptive than neuropathic pain (6.7% and 2.9% respectively). Only one participant (0.96%) reported a fifth painful area (hands). The lower limbs below the level of SCI were the most common location of pain in P1 and P2 (39.4% and 10.6% respectively). Most participants described their pain as burning in P1 (32.7%), joint aches in P2 (11.5%) and P3 (4.8%), and muscular aches in P4 and P5 (1.9% and 0.1% respectively). The mean pain severity (SD) was 6.7 (2.3) for P1; 2.4 (3.0) for P2; 0.6 (1.7) for P3; and 0.2 (0.9) for P4. The one participant who reported P5 in the hands rated the severity of the pain as 3.5/10 on the NRS.

### Type of pain medication used by the participants

Fifty-four participants (51.9%) who reported pain were not on any pain medication. The 50 participants (48.1%) who used pain medication had a mean (SD) age of 38.5 (10.6) years, and the mean age (SD) when they were injured was 32.46 (10.8), while the mean years living with SCI was 6.0 years. Similar to the overall group (*n* = 122), most of the participants who used pain medication were black (84%), male (66%), had complete injuries (74%) at the T6–T12 neurological levels (80%), and had neuropathic pain (82%). They resided with their own families (48%), were unemployed (58%), and relied on the government disability grant (50%).

Table 1 depicts the type of medication the 50 participants used to manage their pain, as well as the type of pain the medications were used for. Opioids were mainly used in combination with other types of medications such as anticonvulsants and NSAIDs (*n* = 22, 44.0%), followed by non-opioid combination medication (*n* = 9, 18.0%), and opioids in isolation were the third most common type of medication used (*n* = 8, 16.0%), and were used for neuropathic pain in all eight instances.

**TABLE 1:** Pain and medication descriptions (*N* = 50).

Types of pain medication			*N*	%	Type of pain			
					Nociceptive		Neuropathic	
					*n*	%	*n*	%
Type of pain medication	Anticonvulsants		6	12.0	1	16.7	5	83.3
	Paracetamol		3	6.0	2	66.7	1	33.3
	Non-steroidal anti-inflammatories (NSAIDs)		2	4.0	1	50.0	1	50.0
	Opioids		8	16.0	0	0.0	8	100.0
	Non-opioid combination (*n* = 9, 18.0%)	Paracetamol + anticonvulsants	3	6.0	0	0.0	3	100.0
		Paracetamol + NSAIDs	5	10.0	2	40.0	3	60.0
		NSAIDs + anticonvulsants + Benzodiazepine	1	2.0	0	0.0	1	100.0
	Opioid combination (*n* = 22, 44.0%)	Opioids + Paracetamol	5	10.0	2	40.0	3	60.0
		Opioids + Paracetamol + NSAIDs	3	6.0	0	0.0	3	100.0
		Opioids + Paracetamol + NSAIDs + anticonvulsants + antidepressants (SSRI)	1	2.0	0	0.0	1	100.0
		Opioids + Paracetamol + NSAIDs + tranquiliser	1	2.0	1	100.0	0	0.0
		Opioids + Paracetamol + anticonvulsants	1	2.0	0	0.0	1	100.0
		Opioids + NSAIDs	2	4.0	0	0.0	2	100.0
		Opioids + NSAIDs + antidepressant (tricyclic)	1	2.0	0	0.0	1	100.0
		Opioids + anticonvulsant	1	2.0	0	0.0	1	100.0
		Opioids + anticonvulsant + antidepressant (tricyclic)	3	6.0	0	0.0	3	100.0
		Opioids + anticonvulsant + antidepressant (SNRI)	1	2.0	0	0.0	1	100.0
		Opioids + antidepressant (tricyclic)	2	4.0	0	0.0	2	100.0
		Opioids + anticonvulsant + antidepressant (tricyclic) + antidepressant (SSRI) + Paracetamol	1	2.0	0	0.0	1	100.0

NSAIDS, non-steroidal anti-inflammatory drugs; SNRI, serotonin-norepinephrine reuptake inhibitor; SSRI, serotonin selective reuptake inhibitor.

### Pain medication questionnaire

The mean (SD) PMQ score was 29.82 (12.01), with the minimum score being zero and 57 being the maximum score out of a possible 104 points. The findings revealed that 22 of the 50 participants (44%) who used pain medication scored ≥ 30, which is indicative of serious aberrant drug-taking behaviours. Twelve participants (24%) scored between 25 and 29, suggesting a risk of future problematic use. Table 2 shows the responses from the participants who used pain medication. A total of 32% of the participants reported that their doctor did not spend enough time talking to them about the prescribed medication, 62% would not mind changing to new medication if recommended by their doctor, and 38% agreed that trying other interventions as adjuncts to the medication was important.

**TABLE 2:** Pain medication questionnaire descriptive results (*N* = 50).

Items	Disagree		Somewhat disagree		Neutral		Somewhat agree		Agree	
	*n*	%	*n*	%	*n*	%	*n*	%	*n*	%
1. I believe I am receiving enough medication to relieve my pain	8	16	11	22	12	24	9	18	10	20
2. My doctor spends enough time talking to me about my pain medication during appointments	16	32	7	14	6	12	8	16	13	26
3. I believe I would feel better with a higher dosage of my pain medication	15	30	2	4	13	26	10	20	10	20
4. In the past, I have had some difficulty getting the medication I need from my doctor(s)	34	68	4	8	4	8	3	6	5	10
5. I would not mind quitting my current pain medication and trying a new one, if my doctor recommends it	6	12	1	2	6	12	6	12	31	62
6. I have clear preferences about the type of pain medication I need	35	70	3	6	3	6	4	8	5	10
7. Family members seem to think that I may be too dependent on my pain medication	32	64	3	6	6	12	4	8	5	10
8. It is important to me to try ways of managing my pain in addition to the medication	8	16	3	6	6	12	14	28	19	38
	**Never**		**Occasionally**		**Sometimes**		**Often**		**Always**	
9. At times, I take pain medication when I feel anxious and sad, or when I need help sleeping	31	62	5	10	7	14	2	4	5	10
10. At times, I drink alcohol to help control my pain	46	92	1	2	2	4	1	2	0	0
11. My pain medication makes it hard for me to think clearly sometimes	21	42	3	6	10	20	11	22	5	10
12. I find it necessary to go to the emergency room to get treatment for my pain	40	80	1	2	6	12	2	4	1	2
13. My pain medication makes me nauseated and constipated sometimes	30	60	4	8	6	12	4	8	6	12
14. At times, I need to borrow pain medication from friends or family to get relief	42	84	4	8	1	2	2	4	1	2
15. I get pain medication from more than one doctor in order to have enough medication for my pain	42	84	5	10	0	0	1	2	2	4
16. At times, I think I may be too dependent on my pain medication	24	48	7	14	3	6	4	8	12	24
17. To help me out, family members have obtained pain medications for me from their own doctors	48	96	2	4	0	0	0	0	0	0
18. At times, I need to take pain medication more often than it is prescribed in order to relieve my pain	31	62	6	12	8	16	3	6	2	4
19. I save any unused pain medication I have in case I need it later	10	20	1	2	0	2	0	0	39	78
	**Never**		**Occasionally**		**Sometimes**		**Often**		**Always**	
20. I find it helpful to call my doctor or clinic to talk about how my pain medication is working	35	70	3	6	5	10	1	2	6	12
21. At times, I run out of pain medication early and have to call my doctor for refills	25	50	6	12	8	16	6	12	5	10
22. I find it useful to take additional medications (such as sedatives) to help my pain medication work better	30	60	8	16	4	8	5	10	3	6
	**1**		**2**		**3**		**4**		**≥ 5**	
23. How many painful conditions do you have?	27	54	15	30	5	10	1	2	2	4
	**Never**		**1 Time**		**2 Times**		**3 Times**		**≥ 4 Times**	
24. How many times in the past year have you asked your doctor to increase your prescribed dosage of pain medication in order to get relief?	33	66	11	22	2	4	2	4	2	4
25. How many times in the past year have you run out of pain medication early and had to request an early refill?	22	44	13	26	6	12	5	10	4	8
26. How many times in the past year have you accidentally misplaced your prescription for pain medication and had to ask for another?	45	90	3	6	1	2	1	2	0	0

The authors found positive correlations between the total PMQ score with pain severities of the two most painful areas namely P1 (*r* = 0.368, *p* = 0.009), and P2 (*r* = 0.300, *p* = 0.035). A one-way ANOVA test revealed significant differences in pain severity (*F* [2, 47] = 5.471, *p* = 0.007). Post-hoc Scheffe tests showed that participants who exhibited serious aberrant drug-taking behaviours (scored ≥ 30) experienced more pain intensity (P1) than those who scored ≤ 25 (Table 3).

**TABLE 3:** Group statistics for P1 severity, and pain medication questionnaire categories.

Item		*N*	Mean	SD	SE	95% Confidence interval for mean		Minimum	Maximum
						Lower bound	Upper bound		
P1 severity	< 25	16	6.594	2.1542	0.5386	5.446	7.742	2.0	10.0
	25–29	12	7.125	1.7468	0.5042	6.015	8.235	4.0	9.5
	≥ 30	22	8.477	1.5467	0.3298	7.791	9.163	5.0	10.0
	Total	50	7.550	1.9646	0.2778	6.992	8.108	2.0	10.0

SD, standard deviation; SE, standard error.

Simple linear logistic regression was conducted to determine factors that predicted PMM. The overall model for the type of medication explained 14.9% of the variance, which was revealed to be statistically significant, F (1, 48) = 8.418, *p* = 0.006. The type of medication was confirmed to be a significant predictor of PMM (Beta = 0.386, *p* < 0.01) (Table 4). Pain severity (P1) was also confirmed to be a significant predictor of PMM (Beta = 0.368, *p* < 0.01), with the overall model explaining 13.6% of the variance (F [1, 48] = 7.526, *p* = 0.009).

**TABLE 4:** Simple linear regression to determine the influence of the type of pain on pain medication misuse.

Model	Unstandardised coefficients		Standardised coefficients	*t*	Sig.	95.0% confidence interval for B	
	B	SE	Beta			Lower bound	Upper bound
Type of medication	0.896	0.309	0.386	2.901	0.006	0.275	1.517
P1 severity	2.250	0.820	0.368	2.743	0.009	0.601	3.898

SE, standard error; Sig., significance.

## Discussion

This study’s overall demographic results are similar to the current literature and underpin the dominance of SCI in the young population, especially males (Brinkhof et al. 2016; Gabbe & Nunn 2016).

Similar to the literature, this study found pain to be mainly neuropathic and occurring in the lower limbs below the level of injury (Adriaansen et al. 2013; Varghese et al. 2020). This study found higher serious aberrant drug-taking behaviour (44%) in the 50 participants who used pain medication, in contrast to Clark et al. (2017) and Krause et al. (2015) who found it to be only 17.6% and 26% respectively. Clark et al. (2017) further found that PWSCI who are at risk of misusing pain medication are those who experience greater pain severity and interference, similar to the findings of the current study and those by Jamison, Link & Marceau (2009). In this study, PWSCI who had serious aberrant drug-taking behaviour had more severe pain than those who were not at risk of PMM.

Opioids were the most common type of medication participants in this study used to manage pain, despite neuropathic pain being the most reported type of pain. Anticonvulsants are the recommended first-line pharmacological intervention for neuropathic pain (Davari et al. 2020).

Prescription of opioids to manage pain is common. However, there has been growing contention among HCPs regarding the prescription of opioids because of their harmful effects (Huber et al. 2016). Opioids are infamous for causing dependency and the risk of fatal overdoses is high in an already vulnerable population group such as PWSCI with pain who use pain medication for a prolonged period (Graupensperger et al. 2019; Hand, Krause & Simpson 2018).

Graupensperger et al. (2019) noted that PWSCI have 7.97 greater odds of developing opioid misuse than their able-bodied counterparts. There is already an increasing rate of overdose deaths in the able-bodied population because of opioid analgesics over the past decade, with an estimated 3–78% of able-bodied people with chronic pain misusing prescribed pain medications (Clark et al. 2017). World Health Organization (WHO 2021) reports that more than 70% of approximately 500 000 deaths are related to opioid use, with overdoses accounting for more than 30% of the deaths. Apart from the risk of misuse, common side-effects of opioid use include dizziness, gastrointestinal side-effects such as nausea, vomiting, constipation, and drowsiness and dizziness, among others (Huber et al. 2016). Various factors have been identified that place users at higher risk of opioid abuse such as previous opioid abuse, taking high doses of opioids, and taking opioids in combination with psychotropic medications and central nervous system depressants (Huber et al. 2016; WHO 2021).

This study found that the type of medication predicts PMM, and although opioids were commonly used by participants in this study, the findings are not conclusive on the specific type of medication as various opioid combinations were used. It is common practice for multiple pain management medications to be prescribed for PWSCI, over and above the other medications necessary for bladder or bowel care, spasticity, and other comorbidities such as hypertension (Cadel et al. 2020; Kitzman, Cecil & Kolpek 2017). The multiple pharmacological treatments are termed polypharmacy and come with challenges for HCPs as they need to balance the potential benefits and risks of using many medications to address all medical problems (Kitzman et al. 2017). This study found that 29.8% of participants used a combination of prescription drugs just to manage pain, as compared to 57% found by Kitzman et al. (2017) for overall management following SCI. Although acknowledging that appropriate prescription of polypharmacy can improve the well-being of PWSCI, prescribing multiple pharmacological interventions inappropriately may result in an increased risk of negative drug interactions and ultimately have harmful effects on an individual’s health (Brose, Schneck & Bourbeau 2019; Cadel et al. 2020). Dependency on any drug has dire consequences, such as increased risks of falls because of drowsiness and constipation, which may trigger autonomic dysreflexia, a life-threatening syndrome in PWSCI at or above T6 characterised by a spike in high blood pressure (Brose et al. 2019; Eldahan & Rabchevsky 2018).

Although not tested in this study, PWSCI have reported addiction to prescription medication, especially after many years of use (Fogelberg et al. 2017). Healthcare providers should screen their patients for substance abuse disorders before prescribing medication that increases the risk of dependency (Bombardier et al. 2021; Brose et al. 2019).

The findings of this study suggest that HCPs prescribe opioid medication for the general complaint of pain without ascertaining the exact type of pain PWSCI report. Participants in this study reported that their HCPs did not spend enough time explaining the pain medication, nor did they find it useful to call their HCP to talk about how the pain medication is working. The general lack of time by HCPs to properly assess pain and the reduced interest by HCPs in listening to patients’ concerns and offering treatment methods other than medication are also identified in the literature as barriers to pharmacological pain management (Buscemi et al. 2018; Guilcher et al. 2020; Löfgren & Norrbrink 2012). People with SCI trust their HCPs and would quit their current pain medication if a new one was recommended by their HCP. The involvement of HCPs in optimising pain management is paramount, and by reducing opioid prescription, HCPs may not only be reducing the risk of PMM by PWSCI but potentially improve self-efficacy and engagement in physical activity in reducing pain. Self-managing pain is the lowest among PWSCI who are prescribed higher dosages of opioids (Morasco et al. 2017) and by limiting opioid use, PWSCI may gain confidence in their ability to perform the tasks (such as physical exercises) necessary to relieve their pain. This study found that less than half of PWSCI who reported pain used pain medication, suggesting the use of pain medication as the last resort. This is also supported by the majority of the participants finding it important to try other ways of managing their pain in addition to the pain medication. It must be noted that some PWSCI showed good pain medication use behaviour. The majority of PWSCI only used the medication for its intended use, did not drink alcohol to help control their pain, did not take additional sedatives to assist the pain medication, nor did they borrow pain medication from others or get their medication from more than one HCP.

### Strengths and limitations of the study

This study is the first, to the authors’ knowledge, to investigate PMM in the South African context. Recall bias possibly occurred as the participants needed to recall some instances within the past 12 months. To mitigate against information bias, the data were checked for accuracy, and both the positive and negative results of the PMQ were reported to reduce publication bias. Except for one question in the PMQ where 92% of the participants disagreed with using alcohol to help pain relief, this study did not investigate substance use that may have played a role in increasing the risk of PMM. Substance use such as smoking and cannabis use has been linked to PMM (Clark et al. 2017; Tate et al. 2004). Although the study reached its target sample size for the overall aim of investigating the phenomenon of pain in a South African sample, only 50 PWSCI who reported pain used pain medication. A larger sample size of pain medication users may have yielded different results, especially as almost half of the participants with pain did not take any pain medication; therefore, generalisation should be done with caution.

### Study recommendations

The PMQ is among the frequently used assessment questionnaires that are used to identify PWSCI at risk of PMM. This tool can be included in clinical practice and used to identify patients that have a higher risk of abusing pain medication (Clark et al. 2017; Huber et al. 2016). Furthermore, the PMQ can be used to identify PWSCI that should be excluded from opioid treatment. Granted, patients that do not yet exhibit serious drug-taking behaviours may do so in the future with long-term use of pain medication. The authors, therefore, recommend that pain management not only include pharmacological intervention but also appropriate techniques accommodated by the biopsychosocial model, such as pain neuroscience education (Wijma et al. 2016), therapeutic and psychological interventions such as physiotherapy, and cognitive behavioural therapy respectively (Van Straaten et al. 2017; Hearn & Cross 2020).

## Conclusion

Pain after SCI is debilitating and although pharmacological interventions are commonly used, pain relief remains difficult to achieve. There is an evident high rate of PMM, which may lead to long-lasting side effects, dependency, or overdose. Healthcare providers need to assess PWSCI for the potential risk of dependency before prescribing pain medication, and should consider non-pharmacological interventions to manage pain.

## References

[CIT0001] Adriaansen, J.J., Post, M.W., De Groot, S., Van Asbeck, F.W., Stolwijk-Swüste, J.M., Tepper, M. et al., 2013, ‘Secondary health conditions in persons with spinal cord injury: A longitudinal study from one to five years post-discharge’, *Journal of Rehabilitation Medicine* 45(10), 1016–1022. 10.2340/16501977-1207 24096367

[CIT0002] Bombardier, C.H., Azuero, C.B., Fann, J.R., Kautz, D.D., Richards, J.S. & Sabharwal, S., 2021, ‘Management of mental health disorders, substance use disorders, and suicide in adults with spinal cord injury: Clinical practice guideline for healthcare providers’, *Topics in Spinal Cord Injury Rehabilitation* 27(2), 152–224. 10.46292/sci2702-152 34108836 PMC8152173

[CIT0003] Bouhassira, D., Attal, N., Alchaar, H., Boureau, F., Brochet, B., Bruxelle, J. et al., 2005, ‘Comparison of pain syndromes associated with nervous or somatic lesions and development of new neuropathic pain diagnostic questionnaire (DN4)’, *Pain* 114(1), 29–34. 10.1016/j.pain.2004.12.010 15733628

[CIT0004] Brinkhof, M.W., Al-Khodairy, A., Eriks-Hoogland, I., Fekete, C., Hinrichs, T., Hund-Georgiadis, M. et al., 2016, ‘Health conditions in people with spinal cord injury: Contemporary evidence from a population-based community survey in Switzerland’, *Journal of Rehabilitation Medicine* 48(2), 197–209. 10.2340/16501977-2039 26931074

[CIT0005] Brose, S.W., Schneck, H. & Bourbeau, D.J., 2019, ‘An interdisciplinary approach to reducing opioid Prescriptions to patients with chronic pain in a spinal cord injury center’, *Physical Medicine and Rehabilitation* 11(2), 135–141. 10.1016/j.pmrj.2018.09.030 30266347

[CIT0006] Bryce, T.N., Budh, C.N., Cardenas, D.D., Dijkers, M., Felix, E.R., Finnerup, N.B. et al., 2007, ‘Pain after spinal cord injury: An evidence-based review for clinical practice and research. Report of the National Institute on Disability and rehabilitation research spinal cord injury measures meeting’, *Journal of Spinal Cord Medicine* 30(5), 421–440. 10.1080/10790268.2007.11753405 18092558 PMC2141724

[CIT0007] Buscemi, V., Cassidy, E., Kilbride, C. & Reynolds, F.A., 2018, ‘A qualitative exploration of living with chronic neuropathic pain after spinal cord injury: An Italian perspective’, *Disability and Rehabilitation* 40(5), 577–586. 10.1080/09638288.2016.1271023 28054832

[CIT0008] Cadel, L., Everall, A.C., Hitzig, S.L., Packer, T.L., Patel, T., Lofters, A. et al., 2020, ‘Spinal cord injury and polypharmacy: A scoping review’, *Disability and Rehabilitation* 42(26), 3858–3870. 10.1080/09638288.2019.1610085 31068029

[CIT0009] Celik, E. C., Erhan, B., Gunduz, B., & Lakse, E. (2013). ‘The effect of low-frequency TENS in the treatment of neuropathic pain in patients with spinal cord injury’, *Spinal Cord*, 51(4), 334–337. 10.1038/sc.2012.159 23295472

[CIT0010] Clark, J.M., Cao, Y. & Krause, J.S., 2017, ‘Risk of pain medication misuse after spinal cord injury: The role of substance use, personality, and depression’, *Journal of Pain* 18(2), 166–177. 10.1016/j.jpain.2016.10.011 27836813

[CIT0011] Davari, M., Amani, B., Amani, B., Khanijahani, A., Akbarzadeh, A. & Shabestan, R., 2020, ‘Pregabalin and gabapentin in neuropathic pain management after spinal cord injury: A systematic review and meta-analysis’, *Korean Journal of Pain* 33(1), 3–12. 10.3344/kjp.2020.33.1.3 31888312 PMC6944364

[CIT0012] Dijkers, M., Bryce, T., & Zanca, J. (2009). ‘Prevalence of chronic pain after traumatic spinal cord injury: a systematic review’, *Journal of rehabilitation research and development*, 46(1), 13–29. 19533517

[CIT0013] Dijkers, M., 2010, ‘Comparing quantification of pain severity by verbal rating and numeric rating scales’, *Journal of Spinal Cord Medicine* 33(3), 232–242. 10.1080/10790268.2010.11689700 20737796 PMC2920116

[CIT0014] Eldahan, K.C. & Rabchevsky, A.G., 2018, ‘Autonomic dysreflexia after spinal cord injury: Systemic pathophysiology and methods of management’, *Autonomic Neuroscience* 209, 59–70. 10.1016/j.autneu.2017.05.002 28506502 PMC5677594

[CIT0015] Fogelberg, D.J., Leland, N.E., Blanchard, J., Rich, T.J. & Clark, F.A., 2017, ‘Qualitative experience of sleep in individuals with spinal cord injury’, *Occupational Therapy Journal of Research: Occupation, Participation and Health* 37(2), 89–97. 10.1177/1539449217691978 PMC5447661 28196449

[CIT0016] Gabbe, B.J. & Nunn, A., 2016, ‘Profile and costs of secondary conditions resulting in emergency department presentations and readmission to hospital following traumatic spinal cord injury’, *Injury* 47(8), 1847–1855. 10.1016/j.injury.2016.06.012 27343134

[CIT0017] Graupensperger, S., Corey, J.J., Turrisi, R.J. & Evans, M.B., 2019, ‘Individuals with spinal cord injury have greater odds of substance use disorders than non-sci comparisons’, *Drug and Alcohol Dependence* 205, 107608. 10.1016/j.drugalcdep.2019.107608 31606588 PMC6921937

[CIT0018] Guilcher, S.J.T., Everall, A.C., Patel, T., Packer, T.L., Hitzig, S.L., Cimino, S.R. et al., 2020, ‘“The strategies are the same, the problems may be different”: A qualitative study exploring the experiences of healthcare and service providers with medication therapy management for individuals with spinal cord injury/dysfunction’, *BMC Neurology* 20(1), 20. 10.1186/s12883-019-1550-9 31941437 PMC6961330

[CIT0019] Guy, S., Mehta, S., Leff, L., Teasell, R., & Loh, E. (2014). ‘Anticonvulsant medication use for the management of pain following spinal cord injury: systematic review and effectiveness analysis’, *Spinal Cord*, 52(2), 89–96. 10.1038/sc.2013.146 24296804

[CIT0020] Hand, B.N., Krause, J.S. & Simpson, K.N., 2018, ‘Dose and duration of opioid use in propensity score-matched, privately insured opioid users with and without spinal cord injury’, *Archives of Physical Medicine and Rehabilitation* 99(5), 855–861. 10.1016/j.apmr.2017.12.004 29307814 PMC8042611

[CIT0021] Hearn, J.H. & Cross, A., 2020, ‘Mindfulness for pain, depression, anxiety, and quality of life in people with spinal cord injury: A systematic review’, *BMC Neurology* 20(1), 32. 10.1186/s12883-020-1619-5 31964353 PMC6971852

[CIT0022] Huber, E., Robinson, R.C., Noe, C.E. & Van Ness, O., 2016, ‘Who benefits from chronic opioid therapy? Rethinking the question of opioid misuse risk’, *Healthcare (Basel, Switzerland)* 4(2), 29. 10.3390/healthcare4020029 27417617 PMC4934582

[CIT0023] Jamison, R.N., Link, C.L. & Marceau, L.D., 2009, ‘Do pain patients at high risk for substance misuse experience more pain? A longitudinal outcomes study’, *Pain Medicine (Malden, Mass.)* 10(6), 1084–1094. 10.1111/j.1526-4637.2009.00679.x 19671087

[CIT0024] Katz, N.P., Adams, E.H., Chilcoat, H., Colucci, R.D., Comer, S.D., Goliber, P. et al., 2007, ‘Challenges in the development of prescription opioid abuse-deterrent formulations’, *Clinical Journal of Pain* 23(8), 648–660. 10.1097/AJP.0b013e318125c5e8 17885342

[CIT0025] Kitzman, P., Cecil, D. & Kolpek, J.H., 2017, ‘The risks of polypharmacy following spinal cord injury’, *Journal of Spinal Cord Medicine* 40(2), 147–153. 10.1179/2045772314Y.0000000235 24970339 PMC5430470

[CIT0026] Krause, J.S., Clark, J.M. & Saunders, L.L., 2015, ‘Pain medication misuse among participants with spinal cord injury’, *Spinal Cord* 53(8), 630–635. 10.1038/sc.2015.42 25777330

[CIT0027] Löfgren, M. & Norrbrink, C., 2012, ‘“But I know what works”-patients’ experience of spinal cord injury neuropathic pain management’, *Disability and Rehabilitation* 34(25), 2139–2147. 10.3109/09638288.2012.676146 22512334

[CIT0028] Mahnig, S., Landmann, G., Stockinger, L. & Opsommer, E., 2016, ‘Pain assessment according to the International Spinal Cord Injury Pain classification in patients with spinal cord injury referred to a multidisciplinary pain center’, *Spinal Cord* 54(10), 809–815. 10.1038/sc.2015.219 26754471

[CIT0029] Mashola, M.K., Korkie, E. & Mothabeng, D.J., 2021, ‘Pain and its impact on functioning and disability in manual wheelchair users with spinal cord injury: A protocol for a mixed-methods study’, *BMJ Open* 11(1), e044152. 10.1136/bmjopen-2020-044152 PMC7789463 33408217

[CIT0030] Mashola, M.K., Korkie, E. & Mothabeng, D.J., 2022, ‘The presence of pain in community-dwelling South African manual wheelchair users with spinal cord injury’, *South African Journal of Physiotherapy* 78(1), 10. 10.4102/sajp.v78i1.1600 PMC8905372 35281780

[CIT0031] Mashola, M.K. & Mothabeng, D.J., 2019, ‘Associations between health behaviour, secondary health conditions and quality of life in people with spinal cord injury’, *African Journal of Disability* 8, 463. 10.4102/ajod.v8i0.463 31309047 PMC6620481

[CIT0032] Morasco, B.J., Yarborough, B.J., Smith, N.X., Dobscha, S.K., Deyo, R.A., Perrin, N.A. et al., 2017, ‘Higher prescription opioid dose is associated with worse patient-reported pain outcomes and more health care utilization’, *Journal of Pain* 18(4), 437–445. 10.1016/j.jpain.2016.12.004 27993558 PMC5376359

[CIT0033] Peduzzi, P., Concato, J., Kemper, E., Holford, T.R. & Feinstein, A.R., 1996, ‘A simulation study of the number of events per variable in logistic regression analysis’, *Journal of Clinical Epidemiology* 49(12), 1373–1379. 10.1016/s0895-4356(96)00236-3 8970487

[CIT0034] Pilusa, S.I., Myezwa, H. & Potterton, J., 2021, ‘Experiences of secondary health conditions amongst people with spinal cord injury in South Africa: A qualitative study’, *South African Journal of Physiotherapy* 77(1), 1530. 10.4102/sajp.v77i1.1530 33937547 PMC8063775

[CIT0035] SAMHSA, 2016, *Opioid overdose prevention TOOLKIT*, viewed 07 May 2022, from https://store.samhsa.gov/sites/default/files/d7/priv/sma18-4742.pdf.

[CIT0036] Sehgal, N., Manchikanti, L. & Smith, H.S., 2012, ‘Prescription opioid abuse in chronic pain: A review of opioid abuse predictors and strategies to curb opioid abuse’, *Pain Physician* 15(suppl. 3), Es67–Es92. 10.36076/ppj.2012/15/ES67 22786463

[CIT0037] St Marie, B., 2019, ‘Assessing patients’ risk for opioid use disorder’, *AACN Advanced Critical Care* 30(4), 343–352. 10.4037/aacnacc2019931 31951657 PMC7029809

[CIT0038] Tate, D.G., Forchheimer, M.B., Krause, J.S., Meade, M.A. & Bombardier, C.H., 2004, ‘Patterns of alcohol and substance use and abuse in persons with spinal cord injury: Risk factors and correlates’, *Archives of Physical Medicine and Rehabilitation* 85(11), 1837–1847. 10.1016/j.apmr.2004.02.022 15520979

[CIT0039] Tsai, C.Y., Bryce, T.N., Delgado, A.D., Mulroy, S., Maclntyre, B., Charlifue, S. et al., 2021, ‘Treatments that are perceived to be helpful for non-neuropathic pain after traumatic spinal cord injury: A multicenter cross-sectional survey’, *Spinal Cord* 59(5), 520–528. 10.1038/s41393-021-00621-9 33742116

[CIT0040] Van Straaten, M.G., Cloud, B.A., Zhao, K.D., Fortune, E. & Morrow, M.M., 2017, ‘Maintaining shoulder health after spinal cord injury: A guide to understanding treatments for shoulder pain’, *Archives of Physical Medicine and Rehabilitation* 98(5), 1061–1063. 10.1016/j.apmr.2016.10.005 28185640 PMC5532812

[CIT0041] Varghese, J., Anderson, K.D., Widerstrom-Noga, E. & Mehan, U., 2020, ‘A primary care provider’s guide to pain after spinal cord injury: Screening and management’, *Topics in Spinal Cord Injury Rehabilitation* 26(3), 133–143. 10.46292/sci2603-133 33192039 PMC7640913

[CIT0042] Vittinghoff, E. & McCulloch, C.E., 2007, ‘Relaxing the rule of ten events per variable in logistic and Cox regression’, *American Journal of Epidemiology* 165(6), 710–718. 10.1093/aje/kwk052 17182981

[CIT0043] WHO, 2021, *Opioid overdose*, viewed 25 April 2022, from https://www.who.int/news-room/fact-sheets/detail/opioid-overdose.

[CIT0044] Wijma, A.J., Van Wilgen, C.P., Meeus, M. & Nijs, J., 2016, ‘Clinical biopsychosocial physiotherapy assessment of patients with chronic pain: The first step in pain neuroscience education’, *Physiotherapy Theory and Practice* 32(5), 368–384. 10.1080/09593985.2016.1194651 27351769

